# Docking and Molecular Dynamics Simulations Clarify Binding Sites for Interactions of Novel Marine Sulfated Glycans with SARS-CoV-2 Spike Glycoprotein

**DOI:** 10.3390/molecules28176413

**Published:** 2023-09-03

**Authors:** Priyanka Samanta, Sushil K. Mishra, Vitor H. Pomin, Robert J. Doerksen

**Affiliations:** 1 Department of BioMolecular Sciences, School of Pharmacy, University of Mississippi, University, MS 38677-1848, USA; psamanta@go.olemiss.edu (P.S.); sushil@olemiss.edu (S.K.M.); vpomin@olemiss.edu (V.H.P.); 2 Research Institute of Pharmaceutical Sciences, School of Pharmacy, University of Mississippi, University, MS 38677-1848, USA

**Keywords:** cryptic binding sites, glycosaminoglycans, molecular docking, molecular dynamics, binding free energy, SARS-CoV-2 spike glycoprotein

## Abstract

The entry of SARS-CoV-2 into the host cell is mediated by its S-glycoprotein (SGP). Sulfated glycans bind to the SGP receptor-binding domain (RBD), which forms a ternary complex with its receptor angiotensin converting enzyme 2. Here, we have conducted a thorough and systematic computational study of the binding of four oligosaccharide building blocks from novel marine sulfated glycans (isolated from *Pentacta pygmaea* and *Isostichopus badionotus*) to the non-glycosylated and glycosylated RBD. Blind docking studies using three docking programs identified five potential cryptic binding sites. Extensive site-targeted docking and molecular dynamics simulations using two force fields confirmed only two binding sites (Sites 1 and 5) for these novel, highly charged sulfated glycans, which were also confirmed by previously published reports. This work showed the structural features and key interactions driving ligand binding. A previous study predicted Site 2 to be a potential binding site, which was not observed here. The use of several molecular modeling approaches gave a comprehensive assessment. The detailed comparative study utilizing multiple modeling approaches is the first of its kind for novel glycan–SGP interaction characterization. This study provided insights into the key structural features of these novel glycans as they are considered for development as potential therapeutics.

## 1. Introduction

The COVID-19 pandemic caused by severe acute respiratory syndrome coronavirus 2 (SARS-CoV-2) created major health issues for the global population. The urgent need to mitigate the negative impacts of the COVID-19 disease on human health led to global efforts in the field of drug discovery. An investigation on how SARS-CoV-2 enters human cells revealed that the virus’ surface spike glycoprotein (SGP) acts as a mediator by binding to human angiotensin converting enzyme 2 (hACE2) receptor using the SGP receptor binding domain (RBD) [1,2,3,4]. Cells are surrounded by a complex mixture of glycans and glycoconjugates, known as the glycocalyx, which acts as a physical barrier to slow the entry of viruses and other infectious organisms but which can also be involved in signaling and providing co-receptor molecules to enable the entry of the microorganisms [5]. To mediate host cell entry, viruses use attachment factors such as heparan sulfate (HS) to facilitate initial interaction with the host cells. HS is a sulfated glycan classified as a glycosaminoglycan (GAG). GAGs are negatively charged polysaccharides endowed with both signaling and therapeutic properties [6,7,8,9,10,11,12]. The host HS–viral protein interaction has also been identified as a crucial event in many other viruses, including parainfluenza [13], human immunodeficiency virus [14,15], and herpes simplex virus [16]. Seeing the importance of the interaction between HS and SGP to enhance virus–host interaction, efforts have been made to disrupt the intermolecular SGP–hACE2 complex using exogenous heparin (a GAG type related to HS), its derivatives, or glycomimetics. It is thought that the disruption of that complex can impede the entry of the SARS-CoV-2 virus into the host cells, thereby presenting an anti-SARS-CoV-2 effect [17,18,19,20,21,22,23,24]. Heparin has long been known as a highly potent anticoagulant. However, some patients experience heparin-induced thrombocytopenia and bleeding [21,25,26,27].

Our recent study using marine sulfated glycans (MSGs) showed significant anti-SARS-CoV-2 activity mediated through the competitive inhibition of the SGP RBD–HS binding [21]. Anti-SARS-CoV-2 activities of certain MSGs were even higher than that of the international standard unfractionated heparin (UFH). Our work studied a novel fucosylated chondroitin sulfate (FucCS) isolated from the body wall of the sea cucumber species *Pentacta pygmaea* (PpFucCS), and a known sulfated fucan (IbSF) and the fucosylated chondroitin sulfate (IbFucCS) from another sea cucumber *Isostichopus badionotus*. FucCSs are characterized by the presence of alternating units of D-glucuronic acid (GlcA) and D-*N*-acetylgalactosamine (GalNAc) in their backbones and L-fucose (Fuc) units linked to the C3 position of the GlcA as branches [21,28,29]. The FucCS reported prior to that study have mainly been classified into FucCS type I, containing monofucosyl branches, and/or FucCS type II, containing difucosyl branches [29,30]. The new molecule PpFucCS recently reported by Dwivedi et al. can be classified as a mixture of FucCS type I and type II, characterized by the presence of two types of monofucosyl branches and one difucosyl branch [21]. To avoid any possible thrombocytopenia-related or bleeding side-effects, in addition to being anti-SARS-CoV-2 glycans it is important for the MSGs to be devoid of anticoagulant properties. Regarding anticoagulation activity, the studied MSGs showed lower potency than UFH.

In this study, we have conducted a thorough and systematic computational investigation of the binding of the holothurian sulfated glycan building blocks of PpFucCS and IbSF to the wild type (WT) RBD. We used blind and site-targeted molecular docking to identify and characterize all possible cryptic binding sites (those identified through interactions with ligands) in the SGP RBD for four holothurian sulfated glycan-derived oligosaccharide building blocks: two trisaccharides and one tetrasaccharide derived from PpFucCS and the tetrasaccharide-repeating unit of IbSF. We followed up on the site-targeted docking with molecular dynamics (MD) simulations to see whether the poses found from docking were in fact stable, considering that a more accurate treatment of intermolecular forces is expected with force fields compared to docking scoring functions and that MD simulations can also represent solvent, temperature, and temporal effects. We have used three different docking programs and two different force fields for the MD simulations to discern the most dependable modeling approaches in identifying all possible MSG binding sites and exploring the RBD–glycan interactions. 

## 2. Results

### 2.1. Identification of Potential Binding Sites in SGP RBD 

**Figure 1:**
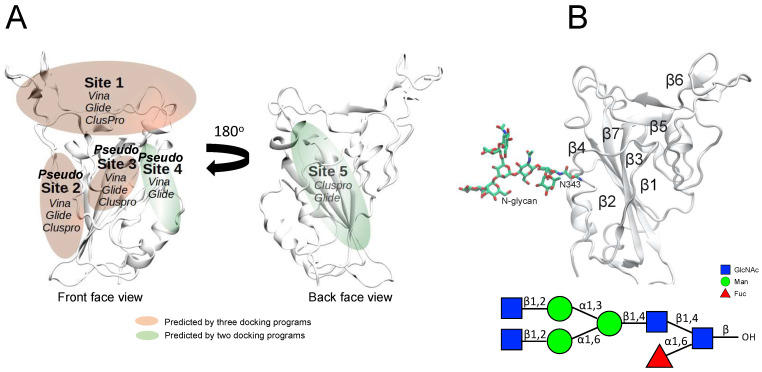
Five binding sites identified by the blind docking protocol using AutoDock Vina, Glide, and ClusPro on the SGP RBD. (**A**) Location of the 2 binding sites and the 3 *pseudo* binding sites. Orange represents the binding sites that were identified by all three docking programs (Sites 1, 2, and 3). Green represents the binding sites that were identified by only two of the docking programs (Sites 4 and 5). (**B**) The β-sheets in the SGP RBD. The SGP RBD was obtained from an ACE2–RBD complex found in the Protein Data Bank (PBD: 6M0J), from which we removed ACE2, a GlcNAc at N343, and water molecules. The structure of the studied N-glycan at residue N343 is shown in SNFG representation.

Several prior studies have found that MSGs exhibit SARS-CoV-2 inhibition by binding to SARS-CoV-2 SGP [21,31]. Here, blind docking was conducted on the SGP RBD using three docking programs, ClusPro protein docking server [32], AutoDock Vina [33], and Glide [34,35,36], with a heparin disaccharide [α-IdoA2S-(1→4)-α-GlcNS6S]. A total of five binding sites (Sites 1–5) were identified for the disaccharide from the blind docking studies (Figure 1A). Glide identified all five binding sites. AutoDock Vina identified only four of the binding sites (Sites 1–4). ClusPro docking identified four of the binding sites (Sites 1, 2, 3, and 5). The various regions of the SGP RBD are labeled in Figure 1B. All of the docking poses obtained from blind docking are shown in Supplementary Figure S1. We chose to use only AutoDock Vina and Glide for site-targeted docking into the sites identified by blind docking, since ClusPro is web-based software with limited adjustability. Starting from the best poses found from site-targeted docking, we performed MD simulations to see whether the docked poses were in fact stable, and concluded that only Sites 1 and 5 were stable, while Sites 2–4 were unstable. 

Previous studies have shown that residue N343 of the SARS-CoV-2 SGP RBD is N-glycosylated with complex-type glycans [37,38,39,40,41]. This glycosylation can play a significant role in enhancing or interfering with RBD–MSG interactions. To investigate the effect of N343 glycosylation on the binding of the MSGs to SGP RBD, we have glycosylated N343 with a core fucosylated biantennary complex-type glycan that has previously been used in several computational studies [42] and performed MD simulations of glycosylated RBD–MSG complexes starting from poses with the MSG docked at Sites 1–5. We then compared the results of the glycosylated RBD–MSG complexes vs. the non-glycosylated RBD–MSG complexes at each potential site.

#### 2.1.1. Site 1 (S1) 

Binding Site 1 was identified by all three docking programs during blind docking (Figure 1A). Previous studies on the holothurian sulfated glycans have shown competitive binding against heparin at this binding site, for both the WT and N501Y SGP mutant [21]. This site contains a crucial residue N501 whose mutation to tyrosine leads to tighter binding to the ACE2 receptor, implicated in higher transmissibility and rates of infection of the mutant form [21,43,44]. In our previous work, we found that the holothurian sulfated glycans bind to the SGP RBD at S1 in a similar mode in the WT and N501Y mutant [21]. Mutation of N501 into Y led to the loss of polar interactions with the hydroxyl of the GalNAc subunit and hence the MSG oligosaccharides were seen to bind in a stronger manner to the WT RBD than to N501Y [21].

**Figure 2:**
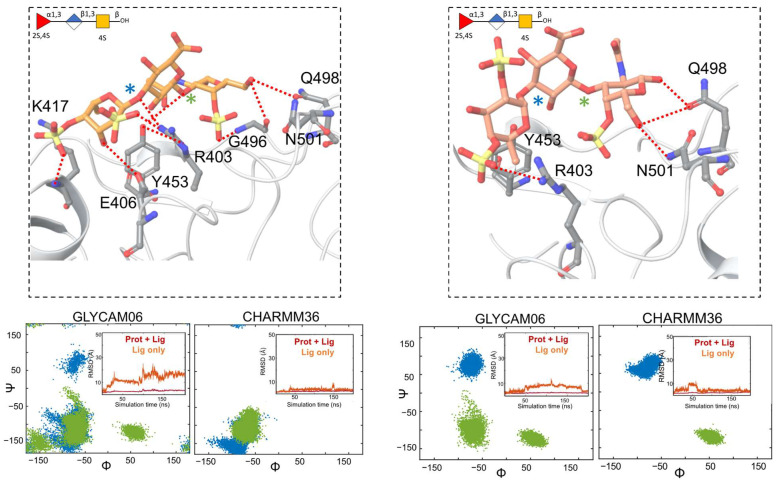
(**Upper panels**) Predicted binding pose of PpFucCS1 at S1 of the non-glycosylated RBD as obtained from Glide (orange; left) and AutoDock Vina (pink; right). The key interacting residues are shown in gray. Dashed lines indicate polar interactions between the RBD residues and PpFucCS1. The MSG is also shown in SNFG representation. (**Lower panels**) Dihedral angle distributions for the glycosidic linkages from the MD simulations, starting from the Glide docked pose (**left**) or from the AutoDock Vina pose (**right**). Each glycosidic linkage is labeled distinctly with blue or green star symbols in the upper panels. Included insert panels show RMSD (in Å) of the heavy atoms of the RBD–MSG complex (Prot + Lig) or of the glycan only (Lig), in red and orange, respectively.

In this work, we have studied the interactions of these glycans at S1 of WT RBD using an alternate docking program, Glide, and compared the results with the previously identified interactions from AutoDock Vina. Glide has previously been used to study docking of GAGs [45,46]. The best scored docking pose for the trisaccharide PpFucCS1 at this binding site is shown in Figure 2. The docked poses for PpFucCS2, PpFucCS3, and IbSF are shown in Supplementary Figure S2. Residue Q498 formed a hydrogen bonding interaction with the O1 hydroxyl of the GalNAc unit. In both AutoDock Vina and Glide docking, interaction with Y453 was maintained, while the COO^−^ of PpFucCS1 was oriented toward the bulk solvent. The charged sulfates of the Fuc unit formed strong ionic interactions with R403. Other strong polar interactions were also observed between the glycans and E406 and K417 in the Glide-obtained best scored docking pose. The AutoDock Vina best scored docking poses for S1 were re-used from Dwivedi et al. [21]. The docking scores from AutoDock Vina and Glide are summarized in Supplementary Table S1. The Glide GScores indicate S1 to be the most favorable of the five binding sites studied here. However, no such conclusion could be made from the AutoDock Vina docking scores. 

**MD simulations of MSGs complexed with the non-glycosylated RBD or the glycosylated RBD at S1.** In order to discern the most reliable approaches for characterizing the protein–MSG interactions and to reduce bias regarding the choice of implemented computational modeling technique in this study, we performed MD simulations of each protein–MSG complex using two different force fields. The top-scored docked poses were subjected to two sets of parallel MD simulations using a combination of: (i) Glycam06 and Amber ff14SB force fields for the MSGs and protein, respectively (referred to as “Glycam06”, below), and (ii) CHARMM36 carbohydrate force field for MSGs and CHARMM36 force field for the protein (referred to as “CHARMM36”, below). To investigate the dynamic behavior of the glycans in their bound state with the SGP RBD, root-mean-square-deviation (RMSD) plots for the simulations for the RBD–MSG complex were prepared (Figure 2 and Figure S2). All simulations at the S1 binding site demonstrated reasonably stable RBD–MSG complexes. This can be seen in Figure 2 and Figure S2, in which the included panels show RBD–MSG RMSD values that remained relatively low throughout the simulation trajectories. In some cases, the ligand showed mobility within the S1 binding site but nevertheless stayed within the site throughout the trajectory. Next, the 3D conformations of the complexes were analyzed by the dihedral angles of their glycosidic linkages found during MD simulation. For the trisaccharide PpFucCS1 complexes obtained from AutoDock Vina docking predictions, the MD simulations showed consistent dihedral angle distributions of the glycosidic linkages for Glycam06 and CHARMM36 force fields (Figure 2). However, Glycam06 showed an additional minimum for the β(1–3) linkage compared to CHARMM36. 

**Figure 3:**
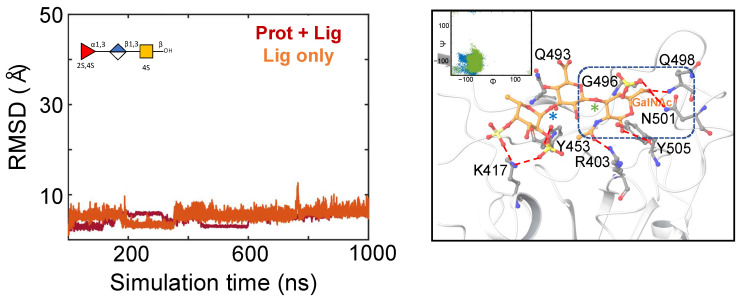
Molecular dynamics simulations of PpFucCS1 at S1 of the glycosylated RBD. (**Left**) RMSD (in Å) of the heavy atoms of the RBD–MSG complex (Prot + Lig) or of the glycan only (Lig), are shown in red and orange, respectively. The glycan is also shown in SNFG representation. (**Right**) Binding pose of PpFucCS1 at S1 of the glycosylated RBD as obtained from MD simulation trajectories (C orange licorice). The included panel shows the dihedral angle distribution for the glycosidic linkages of the dominant conformational form of the glycan. Each glycosidic linkage is labeled distinctly with blue or green star symbols.

We have performed 1 µs MD simulations of the glycosylated RBD–MSG complexes at S1 using the Glycam06 force field. The binding poses of the MSGs during the MD simulations were investigated. During the major part of the simulations, the binding poses of the two trisaccharides (PpFucCS1 and PpFucCS2) were seen to be similar, in which the GalNAc moiety, the reducing end of the glycans, faced toward the sub-pocket containing residue N501 (Figure 3 and Figure S3A). Residue N501 maintained hydrogen-bonding interactions with PpFucCS1 and PpFucCS2 for ~60 and ~70% of the simulation time, respectively. For all three oligosaccharide building blocks from *Pentacta pygmaea* (PpFucCS1, PpFucCS2, and PpFucCS3), residue K417 exhibited an electrostatic interaction with the negatively charged sulfate groups of the terminal fucose moiety, the non-reducing end of the MSGs (Figure 3 and Figure S3A,B). The IbSF also showed interactions with residue N501 throughout the MD simulation (Supplementary Figure S3C). Residue R403 was seen to be involved in interaction with all four MSGs (Figure 3 and Figure S3A), and those interactions were maintained for ~50–70% of the simulation time. No interaction was observed between the MSGs and the distant N-glycan attached to residue N343. It is interesting to note that all four MSGs complexed at S1 of the glycosylated RBD stayed within the binding site through the MD simulations. Hence, Site 1 is a reliable binding site for these compounds.

#### 2.1.2. Site 2 (S2) 

Binding site 2 (S2) was identified by ClusPro, Glide, and AutoDock Vina during the blind docking studies. This site is characterized by interactions of the MSGs with residues at and near the β1-sheet of the RBD (Figure 1). Supplementary Figure S4 shows the top-scored docked pose for PpFucCS1 as obtained from Glide and AutoDock Vina site-targeted docking at S2. In both the cases, the overall docking pose was similar and the same key interactions of the glycan with the SGP RBD were observed.

**MD simulations of the MSGs complexed with the non-glycosylated RBD and the glycosylated RBD at S2.** MD simulations of the non-glycosylated RBD–MSG complexes showed significant statistics of non-stable complexes at this site. The interactions observed in molecular docking were maintained during the beginning part of the MD simulations. However, as the simulation progressed, these RBD–glycan interactions were seen to be broken and, in many cases, the MSGs dissociated from the protein. In some cases, the glycans moved to more favorable binding sites and remained there for the latter part of the simulations. 

Similar to the non-glycosylated RBD, MD simulations of the glycosylated RBD–MSG complexes also showed significant statistics of non-stable complexes at S2, in which the MSGs dissociated from the RBD. No interaction was observed between the N-glycan at residue N343 and the MSGs complexed at this site. Therefore, Site 2 is best classified as a *pseudo* binding site that, when studied with MD, proved to be unstable.

#### 2.1.3. Site 3 (S3) 

Binding site 3 (S3) was identified by ClusPro, Glide, and AutoDock Vina during the blind docking studies. This binding site is characterized by interactions of the sulfated glycans with residues at and near the β3-sheet at the front face of the SGP RBD (Figure 1). 

**MD simulations of the MSGs complexed with the non-glycosylated RBD and the glycosylated RBD at S3.** MD simulations of the RBD–MSG complexes were unstable at this site, in which the MSGs either dissociated completely or separated from the binding site and moved to other regions of the RBD. Thus, Site 3 is merely a *pseudo* binding site.

#### 2.1.4. Site 4 (S4) 

Binding site 4 (S4) was identified by both Glide and AutoDock Vina during the blind docking studies. This binding site is characterized by interactions of the sulfated glycans with residues at and near the β4-sheet of RBD (Figure 1). 

**MD simulations of the MSGs complexed with the non-glycosylated RBD and the glycosylated RBD at S4.** A closer analysis of the MD simulation trajectories showed that the RBD–MSG interactions at S4 were not maintained throughout the simulation and all MSGs dissociated from the binding site of the non-glycosylated RBD. A total of 50% of the glycosylated RBD–MSG complexes at S4 dissociated from the protein during the MD simulations (Supplementary Figure S5). Due to the significant statistics of the non-stable MD simulation of the protein–MSG complexes, we conclude that S4 is, like Sites 2 and 3, a *pseudo*, not real, binding site, both for the non-glycosylated as well as for the glycosylated RBDs.

#### 2.1.5. Site 5 (S5) 

**Figure 4:**
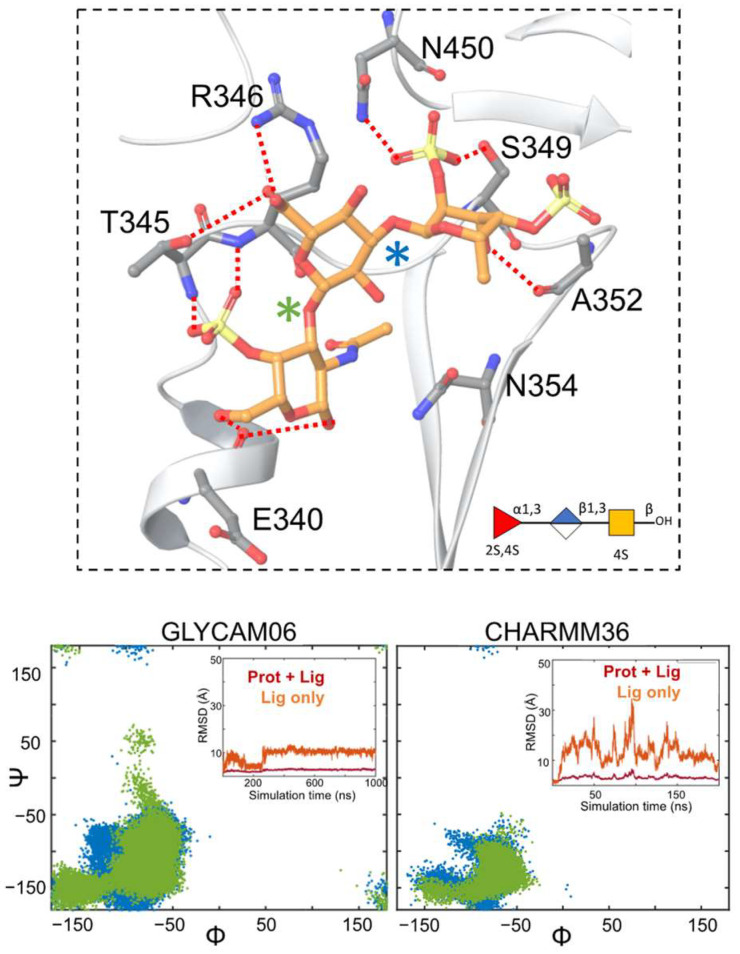
(**Upper panel**) Predicted binding pose of PpFucCS1 at S5 of non-glycosylated RBD as obtained from Glide. PpFucCS1 and the key protein-interacting residues are shown in orange and gray, respectively. Dashed lines indicate polar interactions between the RBD residues and PpFucCS1. The glycan is also shown in SNFG representation. (**Lower panel**) Dihedral angle distribution of glycosidic linkages. Each glycosidic linkage is labeled distinctly with blue or green star symbols. Included panels show the RMSD (in Å) of the heavy atoms of the RBD–MSG complex (Prot + Lig) and of the glycan only (Lig), in red and orange, respectively.

Binding site 5 (S5) was identified by ClusPro and Glide during the blind docking studies. This site is characterized by the interactions of the holothurian sulfated glycans with residues in the β1-, β3-, β4-, and β7-sheets at the back face of the SGP RBD (Figure 1). Previous studies by Clausen et al. have suggested such a heparin/HS-binding site adjacent to the ACE2 binding site [3]. The best Glide-scored pose from site-targeted molecular docking at S5 using Glide is shown in Figure 4 and Supplementary Figure S6. 

The holothurian sulfated glycans were seen to maintain several polar interactions at S5 (Figure 4). The binding mode of each glycan at this binding site was not identical, as could be seen by the involvement of some unique pairs of glycan–protein residue interactions for some of the sulfated glycans (Figure 4 and Figure S6). However, some key protein residues, such as S349, maintained hydrogen bonds with the sulfate groups for three out of the four glycans (Figure 4 and Figure S6A,C). The Fuc unit of the two trisaccharides exhibited hydrogen bonding interactions with A352 (Figure 4 and Figure S6A). The O2 hydroxyl of GlcA of the two trisaccharides interacted with N354. The hydrogen bonding interaction seen between Y351 and the four-sulfate group of the Fuc ring at the non-reducing end of PpFucCS2 was also seen to be maintained in the four-sulfate group of the Fuc ring at the non-reducing end of IbSF (Supplementary Figure S6A,C). The two trisaccharides (PpFucCS1 and PpFucCS2) showed similar overall orientation of binding pose. For PpFucCS1, PpFucCS2, and IbSF, the sub-pocket containing S349 was occupied by the non-reducing end of the glycans. The PpFucCS3 tetrasaccharide, however, showed a different binding pose than the rest, with the reducing end of the glycan occupying this sub-pocket. This could be partly due to the presence of the two negatively charged sulfate groups at the Fuc ring and the larger size of the tetrasaccharide, which enabled it to exhibit strong electrostatic interactions with charged residues such as R355 and K356.

**MD simulations of the MSGs complexed with the non-glycosylated RBD and the glycosylated RBD at S5.** Next, the dynamics of the RBD–MSG complexes were analyzed by parallel sets of MD simulations with different force fields (Figure 4 and Supplementary Figure S6). For PpFucCS1 and PpFucCS3, no significant difference in the distribution of the dihedral angles of the glycosidic linkages was observed by varying the force field for the glycans. For PpFucCS2, one additional minimum for the ⍺(1–3) linkage was observed in the Glycam06 MD simulation. For IbSF, all three ⍺(1–3) linkages showed wider dihedral angle distributions with CHARMM36. The RMSD of the holothurian sulfated glycans showed that all the RBD–MSG complexes were stable during their MD simulation. The CHARMM36 MD simulations showed overall higher fluctuations in the RMSD values than the Glycam06 ones at this site for all four glycans. Regardless, in all cases, the glycans stayed within the binding site region during the MD simulations.

**Figure 5:**
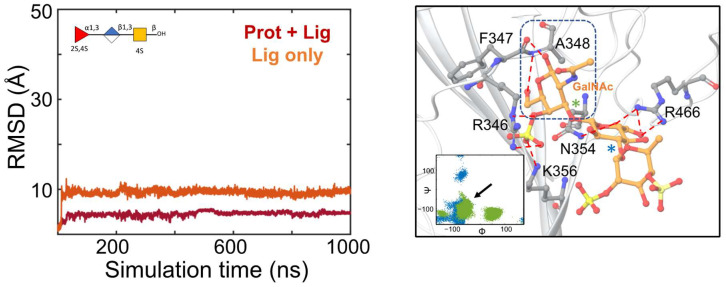
Molecular dynamics simulations of PpFucCS1 at S5 of the glycosylated RBD. (**Left**) RMSD (in Å) of the heavy atoms of the RBD–MSG complex (Prot + Lig) or of the glycan only (Lig), are shown in red and orange, respectively. The glycan is also shown in SNFG representation. (**Right**) Binding pose of PpFucCS1 at S5 of the glycosylated RBD as obtained from MD simulation trajectories (C orange licorice). The included panel shows the dihedral angle distribution for the glycosidic linkages of the dominant conformational form of the glycan. The binding pose orientation shown corresponds to the conformational form of the glycan that is highlighted with an arrow in the included glycosidic distribution plot. Each glycosidic linkage is labeled distinctly with blue or green star symbols.

The interaction modes of the MSGs with the glycosylated RBD at S5 during the MD simulations were analyzed. Figure 5 captures the dominant pose of the PpFucCS1 trisaccharide at S5 during the MD simulation. The pose showed that the GalNAc moiety, the reducing end of PpFucCS1, interacted with the RBD sub-pocket containing residue F347. The dominant conformational form of the RBD-bound PpFucCS1 is marked in the included dihedral angles distribution plot. Electrostatic interactions were observed between the COO^−^ moiety of GlcA and residue R466. Additional electrostatic interactions were observed between the sulfate at the four-position of the GalNAc moiety and residue R346. Similar electrostatic interactions were also observed for the other two oligosaccharides from *Pentacta pygmaea* (PpFucCS2 and PpFucCS3) (Supplementary Figure S7A, pose *b*, and Figure S7B). IbSF exhibited a binding pose in which the sulfate group of the non-reducing end formed similar electrostatic interactions with residue R346 (Supplementary Figure S7C pose *a*). PpFucCS2 and IbSF showed higher fluctuations during the MD simulations than PpFucCS1 and PpFucCS3, which highlighted additional binding poses (Supplementary Figure S7A, poses *a* and *c*, and Supplementary Figure S7C, pose *b*). In all cases, the MSGs stayed within the S5 binding site of the glycosylated RBD. Similar to what was observed for S1, the MSGs complexed at S5 also did not exhibit any interaction with the distant N-glycan attached at residue N343. To summarize, Sites 1 and 5 are reliable binding sites for these compounds.

Conformational analysis of the RBD-bound MSGs at S1 and S5 showed that the MSGs exhibited similar conformation forms when bound to the non-glycosylated RBD vs. the glycosylated RBD (cf. glycosidic linkage dihedral angles distribution plots in Figure 2 and Figure 4 and Supplementary Figures S2 and S6 for the non-glycosylated RBD vs. Figure 3 and Figure 5 and Supplementary Figures S3 and S7 for the glycosylated RBD). This observation was in line with our expectation, as no interaction was observed between the MSGs and the N-glycan at N343 during the simulations.

It was observed that the two sets of MD simulations—using the Glycam06 or CHARMM36 force field—yielded similar results. PpFucCS2, PpFucCS3, and IbSF showed a similar distribution of glycosidic linkage dihedral angles with either of the two force fields, both at S1 and S5 (cf. Supplementary Figures S2 and S6). However, some differences were observed for some pairs of simulations, such as at S1 conformational changes of the bound glycan were observed (Figure 2; left). All four MSGs stayed within the region of their respective binding sites throughout the simulation time and maintained interactions with key pocket protein residues during the simulations, whether for S1 or S5, using either force field. 

The RMSF of the protein residues and the MSG atoms were calculated and are shown in Figure S8. The RMSF of the MSGs showed that the molecules were stable in the binding sites. The RMSF of the protein structures when bound to the MSGs showed that the proteins were stable during the MD simulations. The Rg of the MSGs showed that PpFucCS3 was less compact and exhibited higher conformational flexibility at S1 and S5 than the other MSGs (Figure S9).

### 2.2. ADMET Prediction 

The ADMET properties of the four MSGs were calculated using ADMET Predictor^TM^10.3.0.7 [47]. The wedge plots in Figure S10 compare the molecules’ predicted metabolism and toxicities. Predictions of the four MSGs’ propensities to act as substrates for transporters, namely P-gp, BCRP, OATP1B1, OATP1B3, OCT1, OCT2, OAT1, OAT3, and BSEP, are shown in the left column in Figure S10. All four MSGs were predicted to act as substrates for P-gp (green). The propensities of the four MSGs to act as substrates for the nine CYP isoforms and their predicted intrinsic clearances (CL_int_) are also shown. The MSGs were not predicted to act as substrates for the CYP isoforms and hence were predicted not to exhibit CYP-mediated intrinsic clearance. We also predicted the propensities of the MSGs to act as substrates for the nine UGT isoforms; all MSGs were predicted to be metabolized by UGT2B7 (gray). The three MSGs (PpFucCS1, PpFucCS2, and PpFucCS3) showed lower predicted acute rat toxicity, mutagenesis risk and hERG toxicity than IbSF.

### 2.3. Characterization of the Holothurian Sulfated Glycan Binding Interaction with SGP RBD 

It was observed from the MD simulations that the MSG binding to the SGP RBD is mostly driven by electrostatic interactions with positively charged amino-acid residues (Arg/Lys). Based on the stability of the protein–MSG complexes at S1 and S5, the calculated enthalpic contributions to binding energies from molecular mechanics generalized Born surface area (MM/GBSA) at these two sites of the non-glycosylated and the glycosylated RBDs are listed in Table 1. It is widely known that the additive nature of MM/GBSA calculations is better suited for estimation of the ranking of ligands rather than for obtaining their absolute binding-free energies [48]. Therefore, we have omitted entropic contributions here. All four MSGs showed favorable binding, as could be seen from their negative binding-free energies. IbSF was seen to bind more strongly than the other three sulfated glycans at S1, for both the non-glycosylated as well as the glycosylated RBDs (Table 1). At S1, the MSGs showed the same order of binding to both the glycosylated as well as the non-glycosylated RBDs. For all the four glycans, a large contribution of the electrostatic energy to the binding energy was observed.

**Figure 6:**
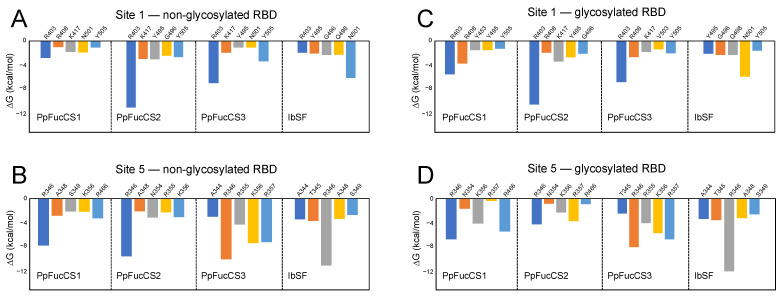
MM/GBSA binding-free energy per-residue decomposition of the five most frequently interacting protein residues at S1 and S5 of (**A**,**B**) the non-glycosylated RBD and (**C**,**D**) the glycosylated RBD.

Per-residue binding-free energy decomposition analysis revealed the contribution of the most interacting protein residues toward the total binding free energy (Figure 6). At S1, selected key interacting residues included R403, Y505, N501, and K417 (Figure 6A). N501 was previously identified to be a crucial residue for binding for these glycans [21]. These key interacting residues were also identified during the docking studies conducted at S1 (Figure 2). At S5, the key residue R346 of both the non-glycosylated or the glycosylated RBD was seen to be interacting strongly with all four glycans (Figure 6B and D). Per-residue-binding free energy decomposition analysis of the glycosylated RBD–MSG complexes at S1 and S5 also showed consistent findings with those of the non-glycosylated RBD (Figure 6). Additionally, the binding-free energy decomposition analysis confirmed that no interaction was observed between the MSGs at S1 or S5 and the N-glycan attached at residue N343. It is important to note that, for each glycan, consistent contributions of the positively charged amino-acid residues (Arg/Lys) to binding was observed. 

**Figure 7:**
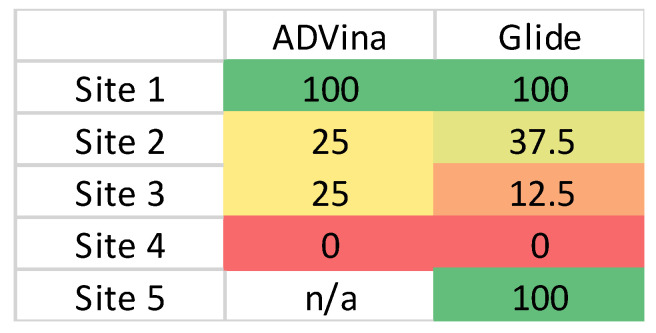
Heat-map showing the percentage of stable non-glycosylated RBD–MSG complexes at each binding site for the MD simulations. Green represents that all simulations at the corresponding site were stable. Red represents when glycans in a specific binding site dissociated, in each case. (n/a: not applicable).

MD simulations provided a detailed investigation of each of the binding sites observed from docking. A total of 16 sets of MD simulations were performed at each site. Regarding the statistical analysis of the stability of RBD–MSG complexes at each site, all complexes at S1 and S5 were stable (Figure 7). S1 has been previously investigated by Kim et al. [18,49], Dwivedi et al. [21], and Maurya et al. [50]. Cao et al. have designed small and stable proteins that can bind tightly at this site to block its interaction with ACE2 [51]. Carino et al. have also studied some potential binding sites on the SGP RBD using naturally occurring and clinically available steroidal agents [52]. Our MD simulations also showed that the glycans bound at S1 were stable, as indicated by their low RMSD values.

It is interesting to note that S2 and S3 were identified by all three docking programs during blind docking. However, RBD–MSG complexes with the MSG at S2, S3, and S4 were highly unstable, as the glycans separated from the sites during the MD simulations. Based on the MD simulations, the glycans remained at Sites 2-–4 for the first ~30–40% of the simulation time before departing. Hence, S2, S3, and S4 should be considered *pseudo*-binding sites based on our study.

S5 had 100% stable MD simulations of the RBD–MSG complexes. It is interesting to note that AutoDock Vina could not predict this binding site in the blind docking runs. Additionally, Glide blind docking predicted a significantly lower population of docked poses at this site (Supplementary Figure S1). During MD, the glycans exhibited several strong polar interactions at this site, making it a highly favorable MSG-binding site.

## 3. Discussion

In this work, we performed a systematic computational analysis on the SARS-CoV-2 SGP RBD binding of four marine oligosaccharides derived from two holothurian sulfated glycans that have anti-SARS-CoV-2 activity: two trisaccharides and one tetrasaccharide derived from the FucCS of *P. pygmaea* (PpFucCS) and the tetrasaccharide building block of the sulfated fucan from *I. badionotus* (IbSF). We considered both non-glycosylated as well as glycosylated forms of the protein. Site-targeted molecular docking identified five binding sites. Using those sites as starting points, MD simulations were performed using two different force fields in order to discern the most reliable approach for binding analysis. A thorough and in-depth analysis pointed out that only two of the potential binding sites, S1 and S5, enabled stable interactions for the negatively charged holothurian sulfated glycans. The computational studies in this work have shown S2, S3, and S4 to be only *pseudo*-binding sites. This suggests that it is unreliable to trust docking programs to predict binding sites for the highly charged sulfated MSG molecules, and therefore a thorough investigation using MD simulations and binding-free energy calculations is recommended to verify MSG binding sites. It should be noted that the nature of MSG binding to proteins is different than the binding of drug-like compounds. Due to the presence of charged groups on MSGs, they do not exhibit a well-defined binding mode in MD simulations [21,53]. Even with higher mobility than glycans in typical RBD–MSG complexes, these MSGs were mobile within the region of their binding sites but did maintain stable interactions in S1 and S5. 

The two force fields used in this study, Glycam06 and CHARMM36, are known to capture glycan–protein interactions and glycan conformational sampling well [54,55]. However, we acknowledge that, in certain cases, force fields may fail to correctly account for particular kinds of protein–ligand interactions. In such cases, further custom force field parameter optimization could be helpful, but such a study is beyond the scope of this work. There are several published reports of the success of utilization of the CHARMM36 force field for calculations on sulfated glycans [55,56,57,58,59,60].

The results of the molecular docking and MD studies in this work are in excellent agreement with some of the previously published reports of the heparin-binding sites on SGP RBD. Site 1 has been previously reported by Dwivedi et al. [21], Kim et al. [18,49], Maurya et al. [50], Mycroft-West et al. [20], Schuurs et al. [53], Cao et al. [51], and Carino et al. [52].

Sites 2 and 5 have previously been reported by Clausen et al. in blind docking of a heparin tetrasaccharide molecule to the SGP RBD [3]. In this study, we have observed S2 to be a *pseudo*-binding site and S5 to be a potential real binding site for the marine sulfated glycans. It is important to note that Clausen et al. investigated sites S2 and S5 by performing a single set of molecular docking calculations. Our work, on the other hand, provides a more comprehensive investigation of each of these sites by the use of three different docking programs as well as using four defined sulfated glycan oligosaccharides with varying sulfation patterns. Additionally, we have performed MD simulations, with two force fields for the glycans, to provide a more reliable approach and better statistics toward confirming the potential binding sites on the RBD. These observations point out the significance of performing MD simulations of protein–MSG complexes in addition to docking, to provide a more accurate understanding of protein–MSG binding. S5 was also identified in recent studies by Kim et al. [61], Paiardi et al. [61,62], and Mycroft-West et al. [20].

This is the first-ever comparative study on the validity of a variety of modeling approaches in SGP–MSG characterization. This study highlights the importance of MD simulations as a follow-up step after molecular docking, in this case to validate the binding of the holothurian sulfated glycans to SGP. Such studies are important in order to be able to provide accurate insight into the potential modifications to the natural product structures that may enhance binding, as they are considered candidates for the development of potential therapeutics.

## 4. Materials and Methods

### 4.1. Preparation of 3D Structures of the Holothurian Sulfated Glycan-Derived Composing Oligosaccharides

**Figure 8:**
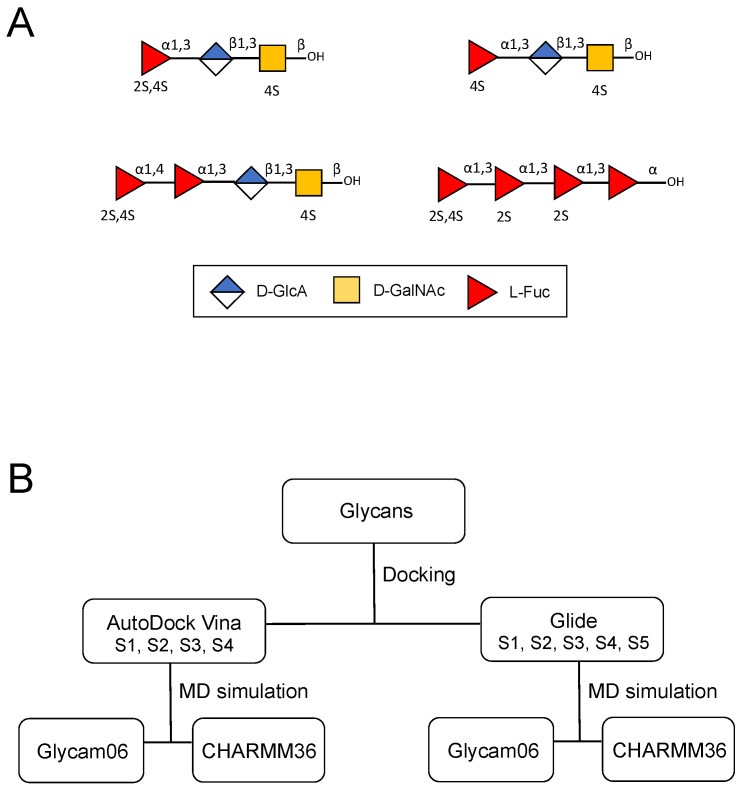
(**A**) Symbol nomenclature for glycans (SNFGs) of the studied MSGs. (**B**) Workflow adopted in the study. Each glycan was subjected to site-targeted docking using AutoDock Vina and Glide. The top-docked pose obtained for each glycan was subjected to MD simulations using the Amber20 package, with the Glycam06 or CHARMM36 force field for the glycans.

The initial structures of the oligosaccharide building blocks of the holothurian sulfated glycans were generated using GLYCAM-Web (glycam.org) and the energy-minimized structures were used for further calculations. The building block oligosaccharides seen in the holothurian sulfated glycans were the following: [α-Fuc2,4S-(1→3)-β-GlcA-(1→3)-β-GalNAc4S] for PpFucCS1, [α-Fuc4S-(1→3)-β-GlcA-(1→3)-β-GalNAc4S] for PpFucCS2, [α-Fuc2,4S-(1→4)-α-Fuc-(1→3)-β-GlcA-(1→3)-β-GalNAc4S] for PpFucCS3, and [α-Fuc2,4S-(1→3)-α-Fuc2S-(1→3)-α-Fuc2S-(1→3)-α-Fuc] for IbSF. The symbol nomenclature for glycans (SNFG) of the studied MSGs are shown in Figure 8A. Depolymerization of the native molecule by mild acid hydrolysis followed by fractionation and NMR structural characterization showed unequivocally that the reading frame of the sulfation pattern of the IbSF tetrasaccharide was [2,4S-2S-2S-nonS] [21]. The overall workflow adopted in this study is summarized in Figure 8B. For docking using AutoDock Vina, AutoDockTools was used to keep polar hydrogens and Gasteiger charges were added to the glycans obtained from GLYCAM-Web [63].

### 4.2. Protein Structure Preparation

The open conformation RBD of one monomer of the SGP was employed in this work. Such a conformation allows for higher accessibility to key interacting residues for glycan binding. The open form of the RBD also allows for accessibility to ACE2 binding [64]. The 3D structure of the WT RBD was obtained from the Protein Data Bank (PDB: 6M0J) [65]. The protein was prepared using the *Protein Preparation Wizard* in Maestro at pH 7.0 ± 2.0. All existing water molecules from the protein PDB were removed. The termini of the protein were capped. This protein structure was used for docking using Glide. For docking using AutoDock Vina, the RBD from the ACE2–RBD complex (PBD: 6M0J) was extracted using VMD1.9.4 [66], AutoDockTools was used to keep polar hydrogens, and Gasteiger charges were added to the protein [63]. The N-glycosylation site N343 is frequently occupied by oligomannose glycans or core-fucosylated glycans [67,68]. Since core-fucosylated N-glycans have been frequently observed in recombinant RBD structures [69], we have used a core-fucosylated biantennary complex-type glycan, a commonly used glycan, for site N343, in some of our computational studies [42] (structure shown in Figure 1B).

### 4.3. Molecular Docking

Blind molecular docking of the MSGs to the RBD was performed using ClusPro [32], AutoDock Vina [33], and Glide [34,35,36,70], followed by site-targeted docking using only AutoDock Vina and Glide. In AutoDock Vina docking, an exhaustiveness of 5 and a seed value of 0 was used. The energy range cut-off was set to 5 kcal∙mol^–1^. For each calculation, 100 and 50 docking poses were obtained for the blind and site-targeted docking, respectively. The top-scored docked pose from site-targeted docking was used as the starting coordinates for the MD simulation. To discern the most reliable docking program for studying these holothurian sulfated glycan-derived oligosaccharide building blocks, in addition to AutoDock Vina docking, the Glide Standard Precision docking protocol in Schrödinger was also used for docking of the sulfated glycans. The glycans were allowed to be flexible and nitrogen inversions and ring conformations were allowed to be sampled, if any. Epik state penalties were added to the docking scores. An energy window of 2.5 kcal·mol^−1^ was used for ring sampling. A distance-dependent dielectric constant of 2.0 was used with 100 minimization steps. The OPLS4 forcefield was used for all the Glide docking calculations [71]. During blind docking, the docking grids used in AutoDock Vina and Glide were made to span the entire RBD and a heparin disaccharide, [α-IdoA2S-(1→4)-α-GlcNS6S], where IdoA and GlcN stand for iduronic acid and glucosamine, respectively, was used as a positive control. The ClusPro protein docking web server was used to perform blind docking studies on the SGP RBD using a fully sulfated heparin tetrasaccharide fragment [32]. A total of 20 models were generated from ClusPro blind docking. The dimensions and the center of the docking box used for site-targeted docking studies with AutoDock Vina and Glide are summarized in Supplementary Table S2. 

### 4.4. SGP–glycan Complex Structure Preparation 

RBD–MSG MD simulations were performed for the complex structures starting from each top-scored docked glycan pose. The systems were prepared using two force fields, Glycam and CHARMM. Disulfide bonds between residue pairs C480–C488, C379–C432, C391–C525, and C336–C361 were added during each system preparation, using *tleap* or CHARMM-GUI, respectively, for Glycam or CHARMM. 

***Glycam.*** The Glycam06 (version j-1) force field was used for the glycans [54]. The force field Amberff14SB was used for the protein [72], and the solvent was represented by the TIP3P water model [73]. The *tleap* program of the Amber20 package was used to prepare the MD input files. All the systems were solvated with an octagonal box having dimensions beyond the protein of 12.0 Å and neutralized with Na^+^ ions. 

***CHARMM.*** The CHARMM36 force field was used for the glycans and the protein [55,74,75]. To generate the glycan force field parameters using the CHARMM36 general force field (CGenFF), the *Ligand Reader & Modeler* module in CHARMM-GUI was used [76,77]. The *Glycan Reader & Modeler* Input Generator in CHARMM-GUI was then used to prepare the RBD–MSG complex systems [78], which were solvated with a rectangular water box with an edge distance of 12.0 Å and neutralized with Na^+^ ions. The CHARMM36 force field was used for the glycans, protein, and water, with WYF parameters for cation–pi interactions and hydrogen mass repartitioning.

### 4.5. MD Simulations

The program package Amber20 was used for all molecular dynamics (MD) simulations, using either the Glycam or CHARMM force fields. For constant temperature simulations, the Langevin thermostat was set to 298 K and periodic boundary conditions were used [79]. A time step of 2 fs was used for the simulations, unless stated otherwise. Non-bonded interactions were kept at a cutoff of 8 Å. The Berendsen barostat was used for constant pressure simulations [80]. Structural analysis and visualizations were performed using Visual Molecular Dynamics (VMD 1.9.4) [66], PyMOL [81], and Maestro [82]. Geometry optimization of the solvent molecules was performed while holding the protein fixed. The systems were equilibrated using a previously published multistep protocol for equilibration of protein–glycan complexes [83]. The system was then heated to 298 K at constant volume for 100 ps, with a time step of 1 fs. Next, 500 ps of equilibration was performed with constant NPT, with protein Cα atoms restrained, and allowing everything else to move. Finally, all atoms were released and equilibrated for 2 ns with constant NPT followed by a production run of 200 ns with constant NPT. For more exhaustive investigation of glycan binding at S5, the RBD–glycan complexes obtained from the Glide best-scored pose were run for an additional 800 ns (yielding a total of 1 µs) of NPT MD simulation, using the Glycam06 force field. MD simulations of the glycosylated RBD–MSG complexes at S1–S5 were performed using the Glycam06 force field and each system was run for 1 µs. MD simulation trajectories were written every 0.02 ns and conformational analysis was performed during the entire production run.

In addition to RMSD calculations, we have also analyzed changes in the radius of gyration (Rg) and root-mean-square fluctuations (RMSF) of the MSGs when bound to the RBD at S1 and S5 using VMD.

### 4.6. Binding-Free Energy Calculations

Each glycan’s binding-free energy (ΔG_binding_) was estimated using the Molecular Mechanics Generalized Born/Surface Area (MM/GBSA) method. In this method, ΔG_binding_ is given by ΔG_binding_ = E_(complex)_ − E_(protein)_ − E_(ligand)_, where ΔG_binding_ is the binding free energy and E_complex_, E_protein_, and E_ligand_ represent the free energies of the RBD–glycan complex, of RBD, and of the glycan, respectively. The implicit solvent model GB^HCT^ (igb = 1) was used for calculating the polar component to the solvation-free energy [84]. The binding-free energies were calculated using the MM/GBSA module in AmberTools20. A total number of 10,000 frames from the first 200 ns of the MD simulations were used to calculate the binding-free energies in each case.

The energy contributions of individual binding site residues to the total binding-free energy of the marine sulfated glycans in complex with the RBD were calculated using the *decomposition module* of the Amber MM/GBSA program [85]. The electrostatic and van der Waals energy contributions to the MM energy together with the polar and non-polar contributions to the solvation-free energy for the five most contributing binding site residues at S1 and S5 are reported.

### 4.7. ADMET Prediction 

The in silico ADMET prediction profiles of the four MSGs were calculated using ADMET Predictor^TM^10.3.0.7 [47].

## Figures and Tables

**Table 1:** Binding-free energies for the four MSG-derived oligosaccharides when bound to the non-glycosylated or the glycosylated RBDs at S1 and S5, calculated using MM/GBSA (kcal^.^mol^−1^). VdWaals, Elec., and EGB represent van der Waals contribution from MM (molecular mechanics), electrostatic energy calculated by MM force field, and electrostatic contribution to the solvation-free energy calculated by GB (generalized Born), respectively. ESurf is the non-polar solvation-free energy. For clarity, complexes obtained from Glide docking that were subjected to Glycam06 force field MD simulations are reported here.

		Non-Glycosylated RBD				
	Ligand	VdWaals	Elec.	EGB	ESurf	∆Total
**S1**	PpFucCS1	−14.23	−273.2	274.3	−1.850	−14.94
	PpFucCS2	−25.54	−303.5	303.2	−3.454	−29.30
	PpFucCS3	−22.51	−314.3	318.2	−2.789	−21.43
	IbSF	−34.51	−267.6	270.6	−3.287	−34.81
**S5**	PpFucCS1	−23.27	−447.9	442.9	−3.012	−31.25
	PpFucCS2	−27.15	−367.6	369.0	−3.214	−28.95
	PpFucCS3	−36.74	−501.6	491.9	−4.417	−50.80
	IbSF	−36.49	−431.4	436.4	−4.046	−35.50
**Glycosylated RBD**						
**S1**	PpFucCS1	−24.92	−304.7	311.1	−3.124	−21.63
	PpFucCS2	−24.36	−326.0	322.6	−3.633	−31.30
	PpFucCS3	−25.01	−322.6	325.0	−3.187	−25.83
	IbSF	−35.03	−266.8	270.3	−3.329	−34.92
**S5**	PpFucCS1	−29.86	−474.5	469.6	−3.548	−38.30
	PpFucCS2	−16.23	−293.8	293.1	−2.097	−18.98
	PpFucCS3	−32.63	−466.3	459.3	−3.974	−43.58
	IbSF	−35.77	−416.5	419.3	−3.844	−36.82

## Data Availability

The data underlying this article are available in the article and in its online supplementary material.

## Supplementary Materials

The following supporting information can be downloaded at: https://www.mdpi.com/article/10.3390/molecules28176413/s1, Figure S1: Overlays of docked poses observed during blind docking studies, performed with a heparin disaccharide on the S-protein RBD prepared from PDB 6M0J; Figure S2: Predicted binding pose from Glide and AutoDock Vina for PpFucCS2, PpFucCS3 and IbSF, bound to S1 of the non-glycosylated RBD SGP RBD; Figure S3: Binding poses of ePpFucCS2, PpFucCS3 and IbSF at S1 of glycosylated RBD as obtained from MD simulation trajectories; Figure S4: Predicted best-docked poses of PpFucCS1 bound at S2, S3 and S4; Figure S5: Heat map showing the percentage of stable glycosylated RBD–MSG complexes at each binding site for the MD simulations conducted using the Glycam06 force field; Figure S6: Predicted binding pose of PpFucCS2, PpFucCS3 and IbSF at S5 of the non-glycosylated RBD as obtained from Glide; Figure S7: Binding poses of PpFucCS2, PpFucCS3 and IbSF at S5 of the glycosylated RBD as obtained from the MD simulation trajectories; Figure S8: The root-mean-square fluctuations (RMSF) of the heavy atoms of PpFucCS1, PpFucCS2, PpFucCS3 and IbSF, at S1 and S5 of the glycosylated RBD during the MD simulation trajectories; Figure S9: The radius of gyration (Rg) of the heavy atoms of the four MSGs at S1 and S5 of the glycosylated RBD during the MD simulation trajectories; Figure S10: Wedge plots showing the predicted metabolism and toxicity profiles for the four MSGs calculated using ADMET Predictor TM10.3.0.7; Table S1: Docking scores of the top scored docked poses for each method and site. ADVina represents AutoDock Vina binding affinities for the glycans. Glide GScores are reported for the Glide docking poses. Values in parentheses denote Glide Emodel values for the best Glide poses; Table S2: Docking box dimensions and box center used for site-targeted docking for AutoDock Vina and Glide.

## Abbreviations

ACE2, angiotensin converting enzyme 2; FucCS, fucosylated chondroitin sulfate; GAG, glycosaminoglycan; GlcA, D-glucuronic acid; GalNAc, D-*N*-acetylgalactosamine; Fuc, L-fucose; IdoA, L-iduronic acid; GlcN, D-glucosamine; HS, heparan sulfate; IbFucCS, FucCS isolated from *Isostichopus badionotus*; IbSF, *I. badionotus*-derived sulfated fucan; MD, molecular dynamics; MSG, marine sulfated glycan; RMSD, Root-mean-square deviation; PpFucCS, FucCS from the sea cucumber *Pentacta pygmaea*; RBD, receptor-binding domain; SF, sulfated fucan; SGP, spike glycoprotein; UFH, unfractionated heparin.
