# Supplementary material for: Docking and Molecular Dynamics Simulations Clarify Binding Sites for Interactions of Novel Marine Sulfated Glycans with SARS-CoV-2 Spike Glycoprotein
Source: Molecules. 2023 Sep 3;28(17):6413. doi: 10.3390/molecules28176413 (PMC10490367; doi:10.3390/molecules28176413)
Supplement: Supplementary file 1 [file molecules-28-06413-s001.zip › molecules-2537221-supplementary.pdf]

Supplementary Data for

Docking and Molecular Dynamics Simulations Clarify Binding Sites for Interactions of Novel Marine Sulfated Glycans with SARS-CoV-2 Spike Glycoprotein

Priyanka Samanta [1], Sushil K. Mishra [1], Vitor H. Pomin [1,2] and Robert J. Doerksen [1,2,*]

[1] Department of BioMolecular Sciences, School of Pharmacy, University of Mississippi, University, MS 38677-1848, USA; psamanta@go.olemiss.edu (P.S.); sushil@olemiss.edu (S.K.M.); vpomin@olemiss.edu (V.H.P.)
[2] Research Institute of Pharmaceutical Sciences, School of Pharmacy, University of Mississippi, University, MS 38677-1848, USA
* Correspondence: rjd@olemiss.edu

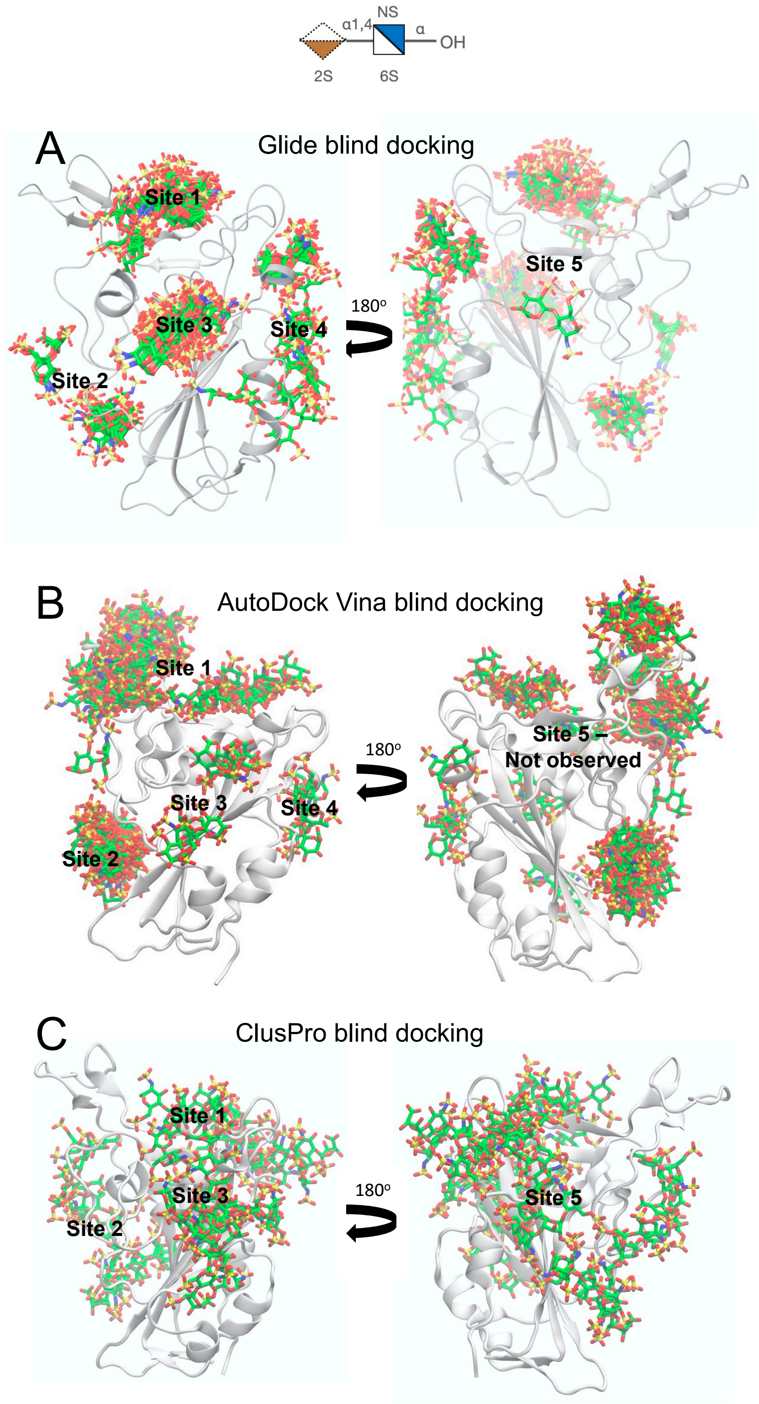

Figure S1. Overlays of docked poses observed during blind docking studies, performed with a heparin disaccharide ([α-IdoA2S-(1→4)-α-GlcNS6S]), on the S-protein RBD prepared from PDB

6M0J. The disaccharide is also shown in SNFG representation. The figure represents all sites identified using (A) Glide, (B) AutoDock Vina, and (C) ClusPro.

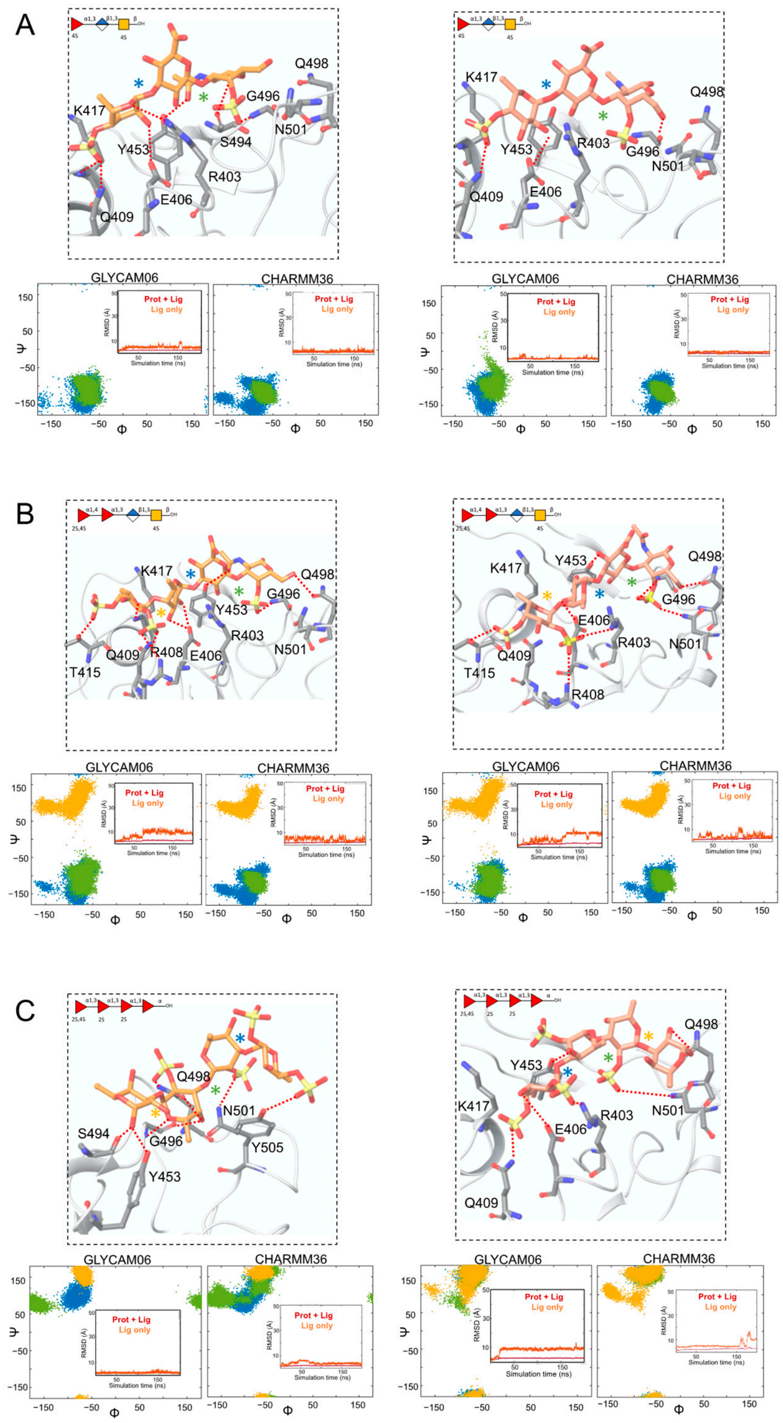

Figure S2. Predicted binding pose from Glide (orange; left panel) and AutoDock Vina (pink; right panel) for (A) PpFucCS2, (B) PpFucCS3 and (C) IbSF, bound to S1 of the non-glycosylated RBD

SGP RBD. The key interacting residues of the protein are shown in gray. Dashed lines indicate polar interactions between the RBD residues and the glycans. Each glycan is also shown in SNFG representation. The lower panels show the dihedral angle distributions for glycosidic linkages. Each glycosidic linkage is labeled distinctly with blue, green or yellow star-symbols. Included insert panels show RMSD (in Å) of the heavy atoms of the protein–glycan complex (Prot + Lig) and of the glycan (Lig) only, in red and orange, respectively.

	PpFucCS1	PpFucCS2	PpFucCS3	IbSF
S1 ADVina	−6.30	−6.94	−7.42	−6.50
S1 Glide	−5.074 (−60.173)	−5.445 (−63.066)	−6.340 (−78.855)	−5.690 (−67.442)
S2 ADVina	−6.2	−6.2	−6.9	−6.8
S2 Glide	−4.293 (−36.310)	−4.390 (−55.264)	−4.744 (−57.397)	−4.593 (−59.869)
S3 ADVina	−6.4	−6.6	−6.6	−6.5
S3 Glide	−4.526 (−36.461)	−3.540 (−22.286)	−3.407 (-40.178)	−4.050 (-51.664)
S4 ADVina	−6.5	−6.6	−7.0	−6.6
S4 Glide	−4.520 (−55.932)	−5.038 (−57.930)	−3.695 (−40.737)	−4.444 (−53.932)
S5 ADVina	n/a	n/a	n/a	n/a
S5 Glide	−4.597 (−60.377)	−4.968 (−57.646)	−4.854 (−58.410)	−4.534 (−59.010)

Table S1. Docking scores of the top scored docked poses for each method and site. ADVina represents AutoDock Vina binding affinities for the glycans. Glide GScores are reported for the Glide docking poses. Values in parentheses denote Glide Emodel values for the best Glide poses.

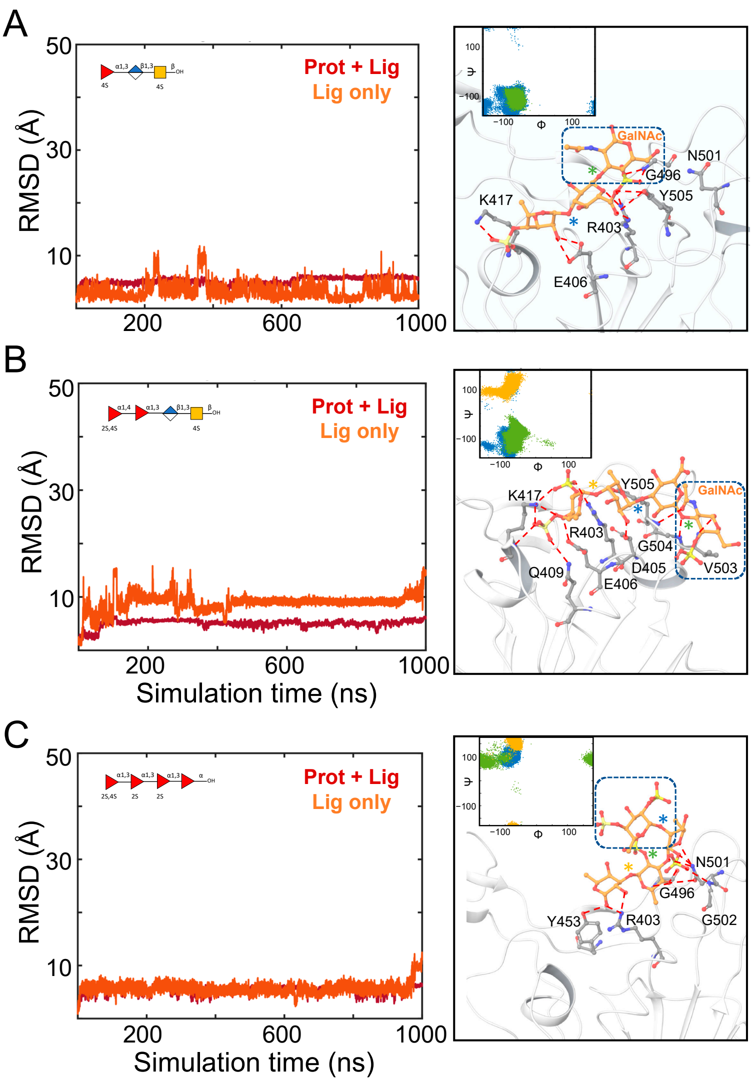

Figure S3. Binding poses of (A) PpFucCS2, (B) PpFucCS3 and (C) IbSF at S1 of glycosylated RBD as obtained from MD simulation trajectories (C orange licorice). The RMSD (in Å) of the heavy atoms of the protein–glycan complex (Prot + Lig) or of the glycan only (Lig) are shown in red and orange, respectively. Each glycan is also shown in SNFG representation. The included panel shows the dihedral angle distribution for the glycosidic linkages of the dominant conformational form of the glycan. Each glycosidic linkage is labeled distinctly with blue or green star-symbols.

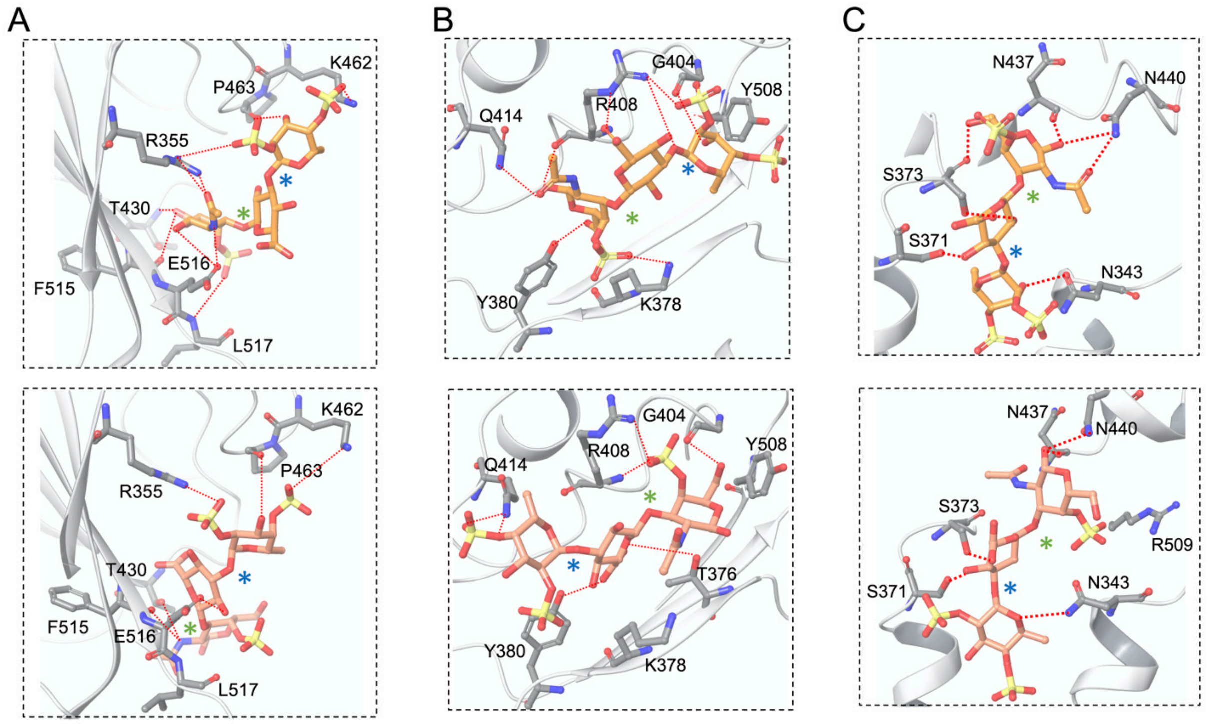

Figure S4. Predicted best-docked poses of PpFucCS1 bound at (A) S2, (B) S3 and (C) S4. PpFucCS1 docked poses using Glide and AutoDock Vina are shown with C orange (top) or pink (bottom), respectively. The key interacting residues are shown with C gray. Dashed lines indicate polar interactions between the RBD residues and PpFucCS1. Each glycosidic linkage is labeled distinctly with blue or green star-symbols. For S3, site-targeted docking predicted opposing docking orientations by AutoDock Vina vs. Glide. However, the key interactions with the same protein residues were maintained in both the cases (Figure S4B). For S4, site-targeted docking studies using Glide and AutoDock Vina predicted similar overall binding orientation. In both cases, the key RBD–MSG interactions were maintained (Figure S4C).

At S3, no significant binding of the MSGs was found in the MD simulations. Such significant statistics of unstable MD simulations of RBD–MSG complexes indicate that the glycans did not

exhibit optimal interactions with protein residues at this site. Similar observations were made for the glycosylated RBD–MSG complexes at S3, in which all four MSGs dissociated from the protein during MD simulations. The MSGs complexed at S3 did not exhibit any interactions with the N-glycan attached at residue N343 (Supplementary Figure S5).

At S4, the MSGs exhibited some interactions with the protein and the mannose/GlcNAc moieties of the N-glycan at residue N343. However, these interactions were not sufficient to keep the MSGs in the binding site, resulting in the dissociation of the oligosaccharide constructs (PpFucCS3 and IbSF) from the glycosylated RBD.

	Glide
Site 1	100
Site 2	0
Site 3	0
Site 4	50
Site 5	100

Figure S5. Heat map showing the percentage of stable glycosylated RBD–MSG complexes at each binding site for the MD simulations conducted using the Glycam06 force field. Green represents that all simulations at the corresponding site were stable. Red represents when glycans in a specific binding site dissociated in each case.

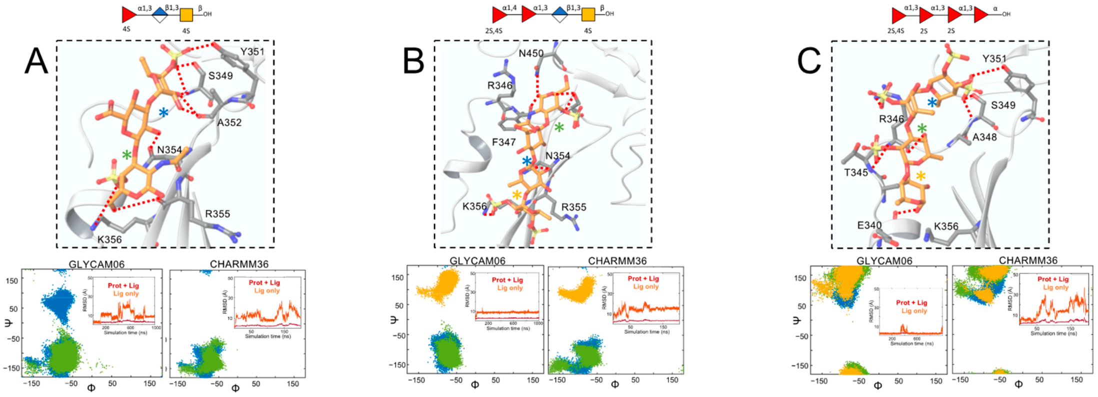

Figure S6. Predicted binding pose of (A) PpFucCS2, (B) PpFucCS3 and (C) IbSF at S5 of the non-glycosylated RBD as obtained from Glide. The glycans and the key interacting residues of the protein are shown in orange and gray, respectively. Dashed lines indicate polar interactions between the RBD residues and the glycans. Each glycan is also shown in SNFG representation. The lower panels show the dihedral angle distributions for the glycosidic linkages. Each glycosidic linkage is labeled distinctly with blue, green or yellow star-symbols. The included insert panels show RMSD (in Å) of the heavy atoms of the whole protein–glycan complex (Prot + Lig) or of the glycan (Lig) only, in red and orange, respectively.

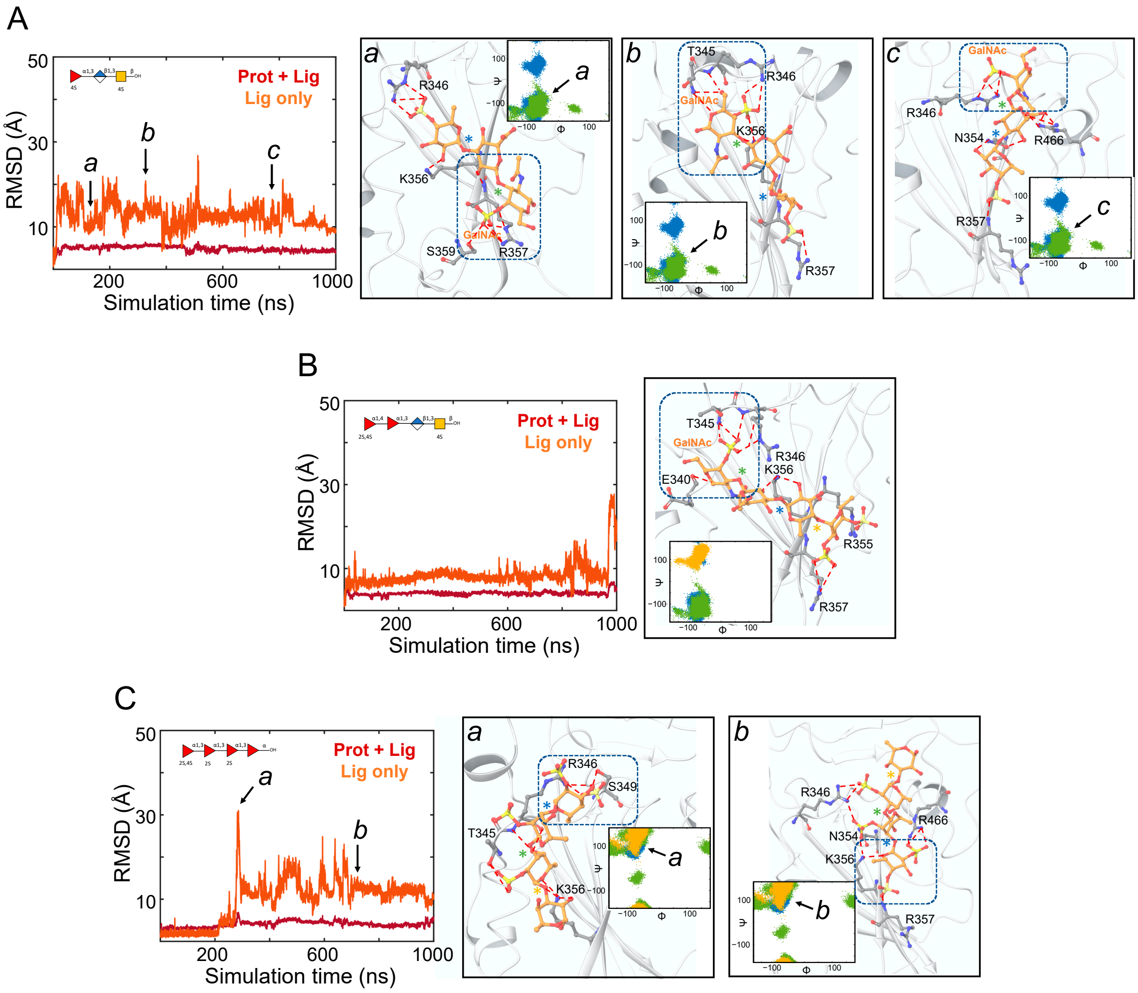

Figure S7. Binding poses of (A) PpFucCS2, (B) PpFucCS3 and (C) IbSF at S5 of the glycosylated RBD as obtained from the MD simulation trajectories. Different binding poses of the glycans found at various times during the MD simulations are shown, labeled as *a, b* or *c* (C orange licorice). The RMSD (in Å) of the heavy atoms of the protein–glycan complex (Prot + Lig) or of the glycan only (Lig) are shown in red and orange, respectively. Each glycan is also shown in SNFG representation. The included panel shows the dihedral angle distribution for the glycosidic linkages of the dominant conformational form of the glycan. Each glycosidic linkage is labeled distinctly with blue or green star-symbols.

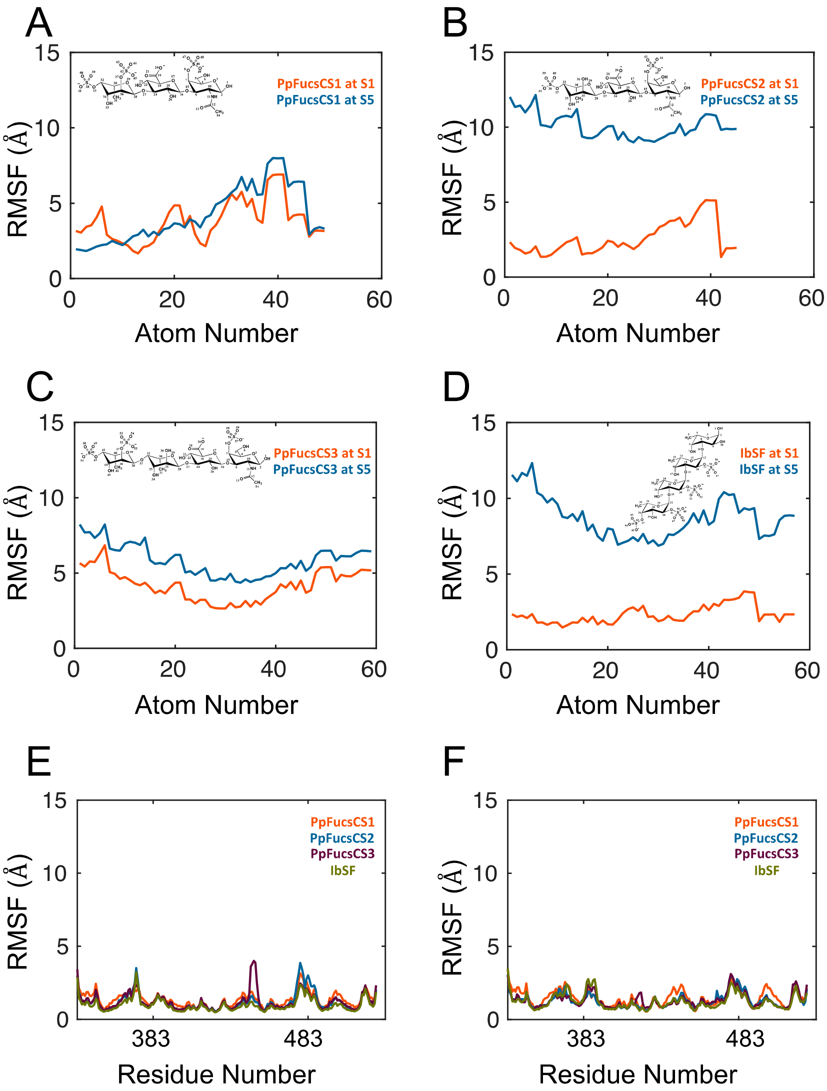

Figure S8. The root-mean-square fluctuations (RMSF) of the heavy atoms of (A) PpFucCS1, (B) PpFucCS2, (C) PpFucCS3 and (D) IbSF, at S1 and S5 of the glycosylated RBD during the MD simulation trajectories. RMSF of the glycosylated RBD C-α residues when the MSGs are bound at (E) S1 and (F) S5. The protein–ligand complex structure at the first frame of the MD simulation was used as a reference in the RMSF calculations.

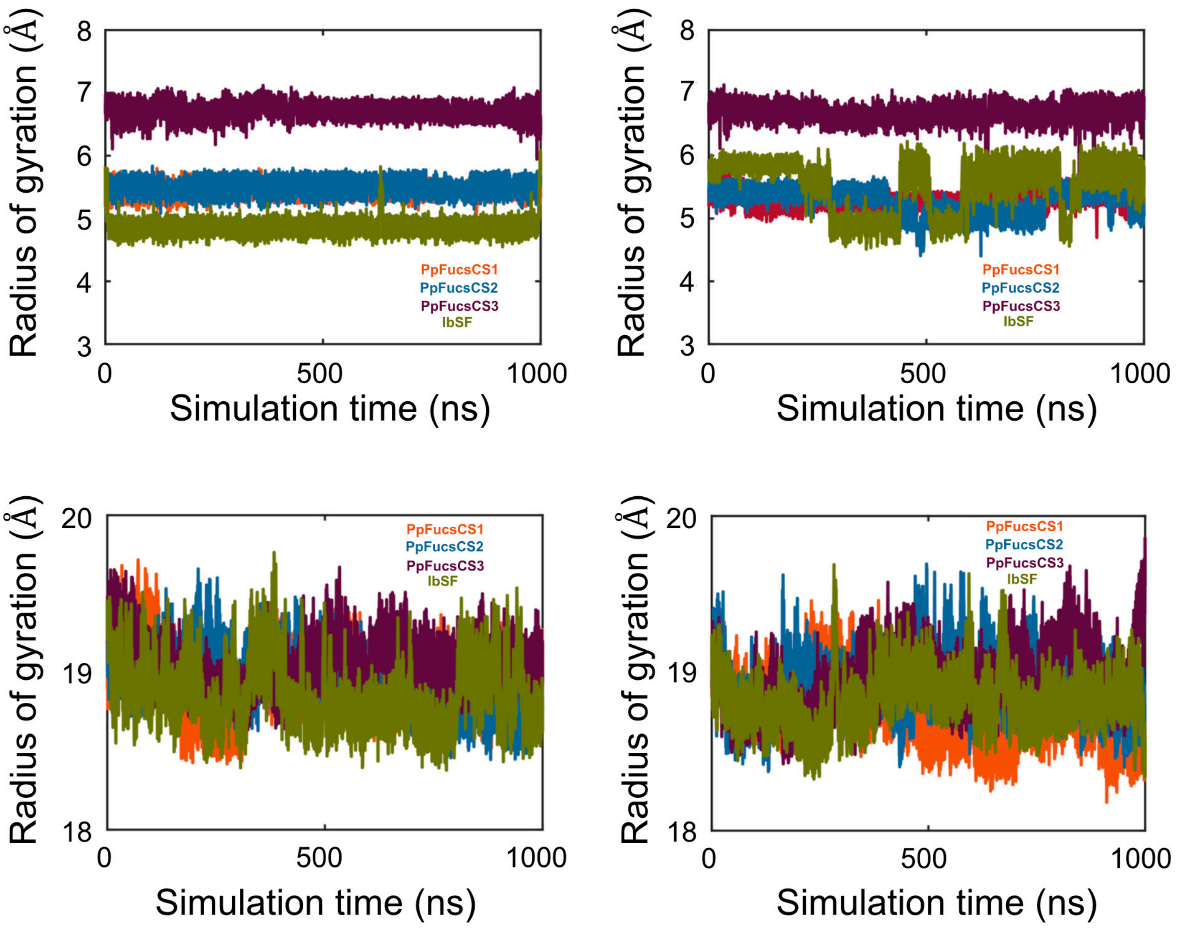

Figure S9. The radius of gyration (Rg) of the heavy atoms of the four MSGs at (A) S1 and (B) S5 of the glycosylated RBD during the MD simulation trajectories. Rg of the glycosylated RBD–MSG complex at (C) S1 and (D) S5.

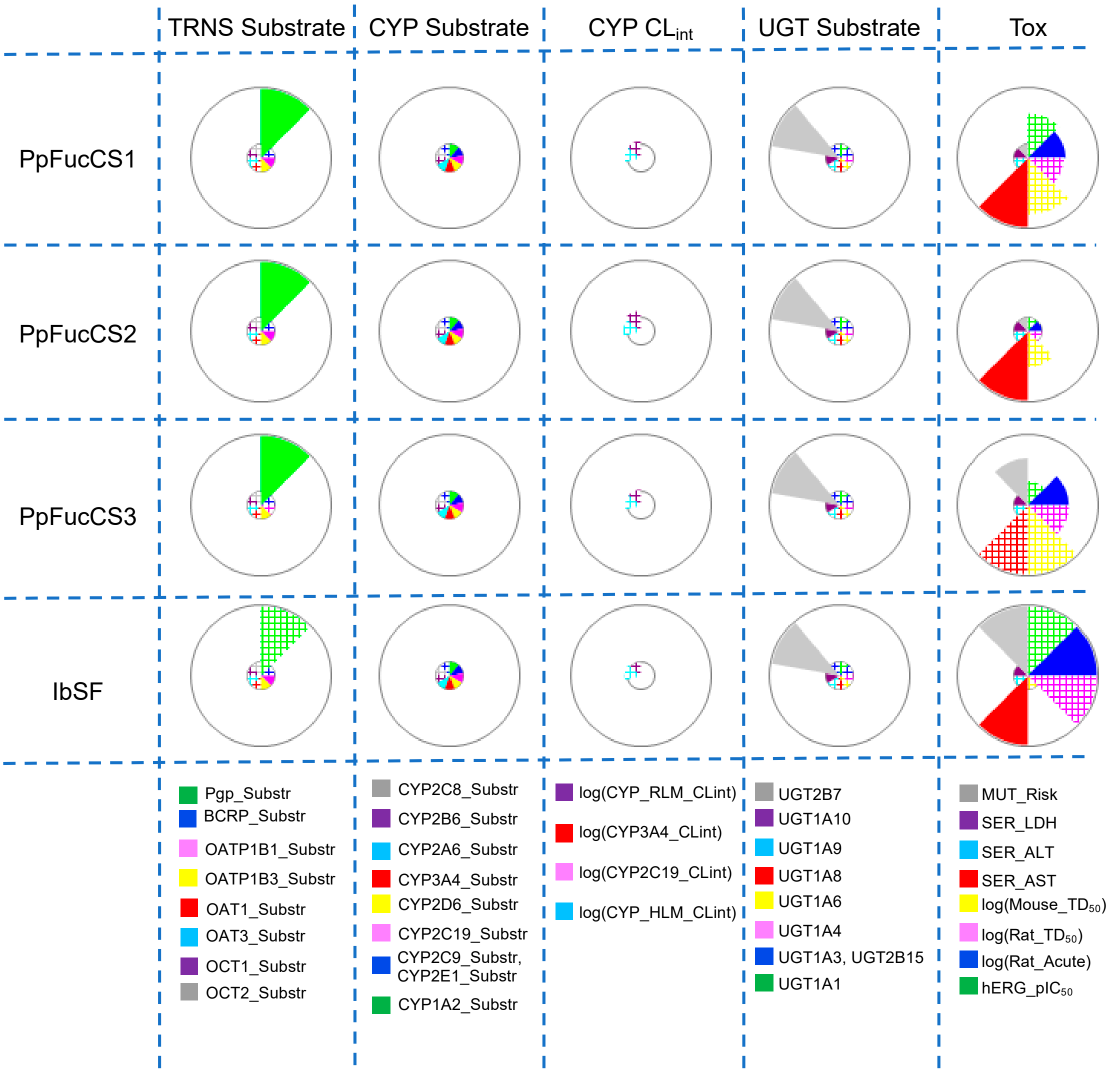

Figure S10. Wedge plots showing the predicted metabolism and toxicity profiles for the four MSGs calculated using ADMET Predictor TM10.3.0.7. (From left to right): substrate propensity for transporters (TRNS Substrate), substrate propensity for the nine CYP isoforms (CYP Substrate); predicted CYP mediated intrinsic clearance (CYP CLint); substrate propensity for the nine UGT isozymes (UGT Substrate); and toxicities (Tox). Cross-hatched wedges represent predictions for out-of-scope compounds.

Binding site	Glide inner box / outer box ($Å^3$)	AutoDock Vina ($Å^3$)	Docking box center
**S1**	35 x 38 x 35 [a] / 40 x 43 x 40 [a]	35 x 38 x 35 [a]	Sidechain O of Y453
**S2**	35 x 35 x 35 / 40 x 40 x 40	35 x 35 x 35	Center of mass of sidechain of F464
**S3**	25 x 25 x 25 / 30 x 30 x 30	25 x 25 x 25	Center of mass of V433
**S4**	27 x 27 x 27 / 32 x 32 x 32	27 x 27 x 27	Centroid of residues S373 and T345
**S5**	25 x 25 x 25 / 30 x 30 x 30	N/A	Centroid of residues R346, R355 and N354

[a] Docking box was made to cover the entire receptor binding motif (RBM) of the S-protein RBD.

Table S2. Docking box dimensions and box center used for site-targeted docking for AutoDock Vina and Glide (N/A for not-applicable).
